# Prairie Dog Optimization Algorithm with deep learning assisted based Aerial Image Classification on UAV imagery

**DOI:** 10.1016/j.heliyon.2024.e37446

**Published:** 2024-09-07

**Authors:** Amal K. Alkhalifa, Muhammad Kashif Saeed, Kamal M. Othman, Shouki A. Ebad, Mohammed Alonazi, Abdullah Mohamed

**Affiliations:** a Department of Computer Science and Information Technology, Applied College, Princess Nourah bint Abdulrahman University, P.O.Box 84428, Riyadh, 11671, Saudi Arabia; b Department of Computer Science, Applied College at Mahayil, King Khalid University, Saudi Arabia; c Department of Electrical Engineering, College of Engineering and Islamic Architecture, Umm Al-Qura University, Makkah, Saudi Arabia; d Department of Computer Science, Faculty of Science, Northern Border University, Arar, 91431, Saudi Arabia; e Department of Information Systems, College of Computer Engineering and Sciences, Prince Sattam bin Abdulaziz University, Al-Kharj, 16273, Saudi Arabia; f Research Centre, Future University in Egypt, New Cairo, 11845, Egypt

**Keywords:** Aerial image classification, Remote sensing, UAV, Prairie dog optimization, Deep learning

## Abstract

This study presents a Prairie Dog Optimization Algorithm with a Deep learning-assisted Aerial Image Classification Approach (PDODL-AICA) on UAV images. The PDODL-AICA technique exploits the optimal DL model for classifying aerial images into numerous classes. In the presented PDODL-AICA technique, the feature extraction procedure is executed using the EfficientNetB7 model. Besides, the hyperparameter tuning of the EfficientNetB7 technique uses the PDO model. The PDODL-AICA technique uses a convolutional variational autoencoder (CVAE) model to detect and classify aerial images. The performance study of the PDODL-AICA model is implemented on a benchmark UAV image dataset. The experimental values inferred the authority of the PDODL-AICA approach over recent models in terms of dissimilar measures.

## 1 Introduction

Unmanned aerial vehicle (UAV) networks link the gap between airborne, spaceborne, and ground-based remote sensing information (RSI). Its lightweight and lowest-price features allow reasonable observations with high time-based and spatial resolutions [1]. The growths in the RSI model and the resultant significant increases in the spatial, temporal, and spectral resolves of remotely detected data and the improved progress in information and communication technologies (ICT) dependent upon storage, data transmission, and integration abilities are remarkably altering the method they observed the Earth [2]. The most vital use of RSI is to observe the Earth, and the core concern in Earth-observing is monitoring the variations in land cover. Aerial imagery classification (AIC) of scenes categorizes the resultant AI into sub-regions by covering several ground things and types of land cover into various semantic sorts [3]. Many actual uses of RSI are accessible, such as urban development, computer cartography, resources management, and AIC, which are very important. Usually, some similar object types of land cover are united amid several forms of scenes [4]. For example, commercial and domestic scenes are two of the foremost sorts of scenes that may contain roads, buildings, and trees. Still, there are dissimilarities in the spatial distribution and density of the three types. So, in aerial scenes, classification entirely depends upon the spatial and structural pattern, which is a complex issue [5].

The standard method is constructing a holistic scene symbol for classification [6]. In the RSI community, bag-of-visual-words (BoVW) is one of the known techniques for scene classification issues. It was made for text analysis that plans a text over the occurrence of words. The bag-of-words (BOW) method was employed with a clustering model to identify imageries of the number of visual words produced by quantizing local features. The BoVW approach is a type of BoW method for image analysis, whereas each image is definite as an order set. Deep learning (DL) methodologies like convolutional neural networks (CNNs) have been generally identified as an excellent technique for frequent computer vision (CV) uses (video or image classification and detection) and have also exposed remarkable outcomes in several uses [7]. Thus, various benefits are reduced from employing DL models in emergency response and disaster organization to recover vital data appropriately, permit superior preparation and response throughout critical situations, and aid in making decision procedures [8]. Past works have proved how DL models can overcome traditional machine learning (ML) techniques with hand-crafted features over the usage of transfer learning (TL), where a pre-trained CNN is applied as feature extraction and one or many layers are inserted on topmost to execute the classification for the novel task [9].

This study presents a Prairie Dog Optimization Algorithm with a Deep Learning Assisted based Aerial Image Classification Approach (PDODL-AICA) on UAV images. The PDODL-AICA technique exploits the optimal DL model for classifying aerial images into numerous classes. In the presented PDODL-AICA technique, the feature extractor process is executed by customizing the EfficientNetB7 approach. The PDODL-AICA technique uses a convolutional variational autoencoder (CVAE) approach to detect and classify aerial images. The performance study of the PDODL-AICA method is carried out on a benchmark UAV image dataset. The contribution of the PDODL-AICA method is as follows:

• The presented PDODL-AICA technique implements the EfficientNetB7 model for feature extraction, utilizing its state-of-the-art model that balances the size and accuracy. EfficientNetB7 is recognized for its capacity to capture convolutional patterns and details in the data, paving the way to a more significant accomplishment in the task of feature extraction
• The hyperparameter tuning of the EfficientNetB7 technique is optimized by employing the PDO method. This optimization approach confirms that the hyperparameters of the EfficientNetB7 model are finely altered to attain optimum accomplishment, improving the comprehensive effectualness and accuracy of the technique
• The approach also incorporates a CVAE model for recognition and classification, allowing for efficient modelling of convolutional data dispersions. The CVAEs method integrates the power of CNNs with variational inference, making them appropriate for tasks needing both recognition and classification
• The novelty of the PDODL-AICA technique is in its novel integration of EfficientNetB7, PDO, and CVAE for advanced feature extraction, hyperparameter tuning, and efficient recognition and classification. This incorporated model addresses several threats in recognition and classification tasks, giving an overall, efficient, and exact outcome

## 2 Literature works

Behera et al. [10] developed a super pixel-aided multiscale CNN architecture to prevent misclassification in complex urban aerial images. The presented structure is a dual-tier DL-based segmentation framework. In the 1st phase, a super-pixel-based simple linear iterative cluster system offers superpixel images with vital contextual data. The 2nd phase includes a multiscale CNN design that employs these data-rich superpixel pictures to remove scale-invariant features. Rahman et al. [11] projected a forest fire detection model reliant on a CNN architecture utilizing a unique fire detection database. The model also employs separate convolution layers for instant fire recognition and usual layers. So creating it appropriate for actual use. Dewangan and Vij [12] present a new structure, like the hybrid CNN and Long Short-Term Memory (HCNN-LSTM), which targets to identify anomalies in farmland utilizing imageries attained from UAVs mechanically. The model uses a CNN for the deep feature extractor, whereas LSTM is used for the recognition task. Behera et al. [13] projected a DL-based technique inclined by the dense units that aid the domain of the system's feed-forward nature, so removing the vanishing gradient issue generally got in deep modern devices. The planned endwise CNN design contains contracting and symmetric growing tracks that exactly remove the global feature to divide the vegetation type from the aerial imageries. Pandey and Jain [14] clarify an innovative conjugated dense CNN (CD-CNN) design named SL-ReLU for intellectual classification. CD-CNN combines data fusion and feature map extractor in combination with classification procedure. Jiskani et al. [15] presented a DL-based model for identifying speeded-over insulator errors in the real. The system is dependent upon the architecture of Resnet 50.

Minu and Canessane [16] proposed an effective DL-based AIC utilizing Inception with Residual Network v2 and a multilayer perceptron (DLIRV2-MLP). UAVs have been used mainly for a range of aerial imagery. The IRV2-based feature extraction has been applied to create a beneficial set of feature vectors. Lastly, the AIC utilizes the resultant feature vector through the MLP model. Samadzadegan et al. [17] developed a new DL-based model for effectively detecting binary sorts of drones and birds. Estimating the planned system with the set image dataset validates superior efficacy equated to present recognition methods. Also, drones are frequently tangled with birds due to their primary and behavioural resemblance. Cheng et al. [18] collectively review DL models covering threats, autoencoder-based, CNN-based, and generative adversarial network-based models. Geetha and Sunitha [19] introduce a Pelican Optimization Algorithm with a Convolutional-Recurrent Hop-Field Neural Network (POA-CRHFNN) model. This method comprises the Gaussian Filter (GF) technique for noise removal and contrast enhancement, ShuffleNetv2 for extraction, and CRHFNN for spatial-temporal dependency capture. In Ref. [20], Archimedes Optimization with DL-based Aerial Image Classification and Intrusion Detection (AODL-AICID) method is presented. This approach employed MobileNetv2, AOA, BPNN, and a stacked Bi-directional LSTM (SBLSTM) model tuned with the Nadam optimizer for extraction, optimization, classification, and detection. Mogaka et al. [21] propose a co-design optimization technique for deploying the EmergencyNet CNN on resource-limited UAVs. This method encompasses channel-wise pruning to mitigate network size and optimize the model. The additive powers-of-two (APoT) quantization is also utilized for additional model compression and enhanced computational effectualness.

## 3 The proposed method

**Fig. 1:**
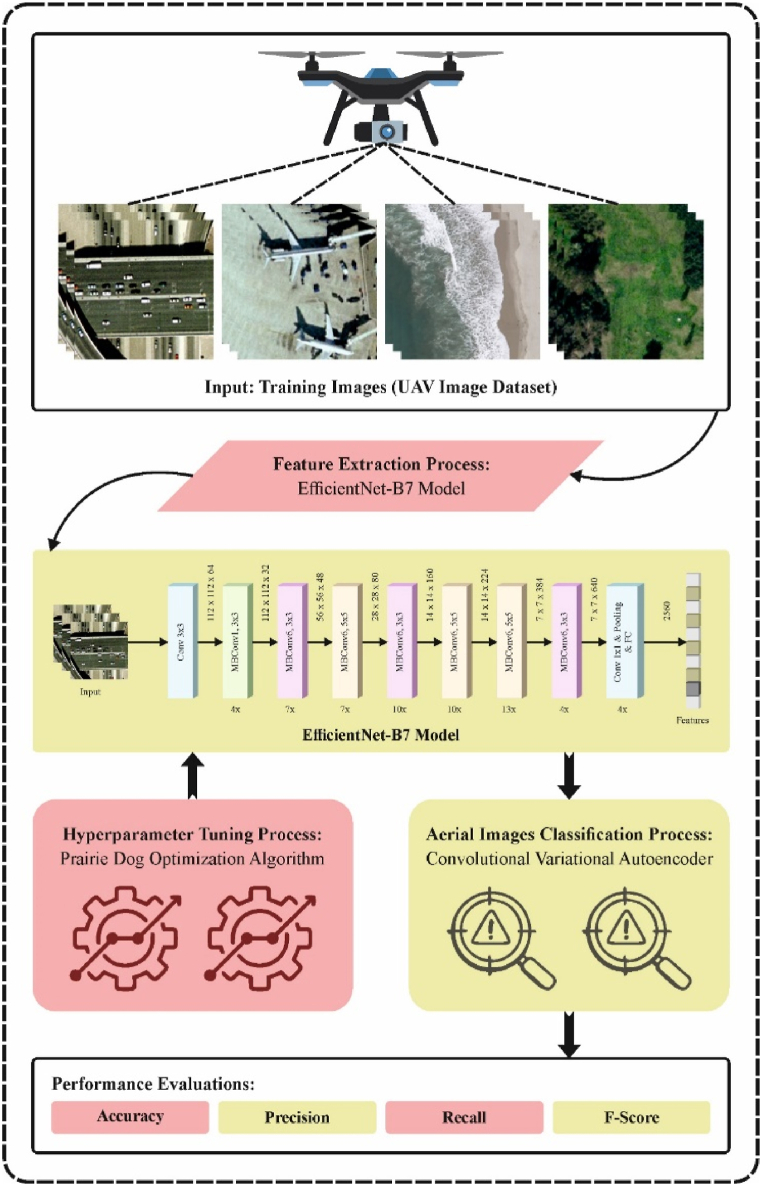
Workflow of PDODL-AICA model.

This research proposes an innovative PDODL-AICA technique for UAV images. The method exploits the optimal DL model for classifying aerial images into manifold classes. It contains an EfficientNetB7-based feature extractor, PDO-based parameter tuning, and CVAE-based classification processes. Fig. 1 portrays the workflow of the PDODL-AICA model.

### 3.1 Feature extraction: EfficientNetB7 model

Using the EfficientNetB7 technique for the feature extractor procedure executes the PDODL-AICA technique. Due to the high upsurge in the convolutional layers, network width, and depth, deep CNN (DCNN) architecture is usually overparameterized, making an architecture computationally expensive and compromising network efficiency [22]. There are tradeoffs between network accuracy and efficiency. Deep networks are well-generalized on testing datasets, and their efficacy in terms of network parameters, model dimensions, inference speed, and flops (floating-point operations per second) increases. Google researchers in 2019 introduced a family of EfficientNet sequences, such as EfficientNetB0 – B7, as a backbone structure that has outperformed many DCNN-based frameworks, namely InceptionV2, ResNet, InceptionV3, DenseNet, and ResNet50 for TL, image classification from segmentation, ImageNet and other issues. This contradicts classical scaling models used by prior research, which include randomly increasing the system's depth, width, and resolution to improve the generalization capability. The CNN can be generalized by the EfficientNet using a secure set of scaling co-efficient over the compound scaling model. Compound scaling is derived from balancing the network size of width w, depth d, and resolution r by ascending it with an endless ratio as shown in Eq. (1).

(1) d=α∅,w=β∅,r=γ∅

In such a way that α.β2.γ2≈2 whereas, α ≥ 1, β ≥ 1, γ ≥ 1. α, β and γ values are defined by the grid search technique. A user-defined parameter defines an upsurge in computation resources to the network is known as ∅. Flops of the convolution network operation are equivalent to d, w2, and r2. That flops will be doubled if the network depth is doubled. At the same time, flops will be increased to four times if the resolution and width are doubled. The increase in flops is based on the relation (α.β2.γ2)∅ such that overall flops are enlarged by 2∅for novel value. The EfficientNet structure has a stem block that follows the seven blocks and a last layer. Every block in EfficientNet contains a variable amount of modules, and the amount of modules upsurge as one profit from EfficientNet-B0 to B7. EfficientNet contains parameters and variable depth. EfficientNetB0 is the simple version of EfficientNet with 5.3M parameters and 237 layers, whereas EfficientNetB7 has 66M and 813 layers. EfficientNet structure exploits MBConv layers, similar to MnasNet and MobileNetV2. Meanwhile, the normalization layer is pre-existing in the stem layer, so no further image standardization is needed as a preprocessing stage, so it takes an input image within the 0–255 range. Here, five variations of pre-trained EfficientNet viz., EfficientNet B0 – B4 support the lung cancer classification from the CT scans. The conditions for choosing EfficientNet variation are based on different variables, like dataset size, the resource accessible for the evaluation and model training, the model depth, the network parameter, and the batch size. EfficientNet-B5 over EfficientNet-B7 are significant differences between EfficientNet with more parameters and deep networks.

### 3.2 Hyperparameter tuning: PDO model

At this stage, the hyperparameter tuning of the EfficientNetB7 technique takes place using the PDO model. PDO is a biologically stimulated optimizer technique stimulated by the four-prairie dog (PD) actions to determine the exploitation and exploration stages of the algorithm [23]. The PD spends their days building new burrows, eating, and keeping present by doing maintenance for it or monitoring for enemies. The PD produces sounds according to the present event that might be an alarm for the predator threat or food source obtainability. They can communicate diverse signals for chasing patterns and different kinds of predators. PDs have dissimilar survival responses dependent on the type of signals attained. Each dog will observe from the entry of the caves in case of a coyote. Each dog will escape into the caves if the translated sound by the predator is human. Hence, dissimilar responses for the predators are applied in the exploitation phase. These survival habitats and communication sounds are the motivation for the PDO technique. While raising their system, certain assumptions were made as scheduled below:

— Every colony creates a group of wards where the entire coterie (CT) occurs in the ward.
— Each PD is a member of CT that has n dogs, whereas m CT forms a colony.
— There is no difference between PDs; they are only divided into different groups.
— The new nutrition supply and anti‐predation are the only sounds of communication.
— The number of caves in the ward arrays from 10 to 100.
— According to the division of the colony into m CT where a similar activity is simultaneously done, the exploitation and exploration are repeated m times.
— The anti‐predation activities, signals transfer, food search, and burrow-creating actions are limited to the dogs living in similar CT.

#### 3.2.1 Initialization

Like other metaheuristic approaches, the PDO technique applies a random initial position for the PDs within the search space. The matrix, as portrayed by Eqs. (2), (3) gives the locations of the PDs within the CT that are part of the colony and the locations of each CT within the colony.

(2) $$CT=\left[\begin{array}{lllll} CT_{1,1} & CT_{1,2} & \cdots & CT_{1,d-1} & CT_{1,d}\\ CT_{2,1} & CT_{2,2} & \cdots & CT_{2,d-1} & CT_{2,d}\\ \vdots & \vdots & CT_{i,j} & \vdots & \vdots \\ CT_{n,1} & CT_{n,2} & \cdots & CT_{n,d-1} & CT_{n,d} \end{array}\right]$$

(3) $$PD=\left[\begin{array}{lllll} PD_{1,1} & PD_{1,2} & \cdots & PD_{1,d-1} & PD_{1,d}\\ PD_{2,1} & PD_{2,2} & \cdots & PD_{2,d-1} & PD_{2,d}\\ \vdots & \vdots & PD_{i,j} & \vdots & \vdots \\ PD_{n,1} & PD_{n,2} & \cdots & PD_{n,d-1} & PD_{n,d} \end{array}\right]$$

here, $CT_{i,j}$ represents the $j^{th}$ dimension of the $i^{th}$ CTs, and PD indicates the $j^{th}$ parameter of the $i^{th}$ PDs in a particular CT, according to Eqs. (4), (5), CT and dog locations are defined correspondingly.

(4) $$CT_{i,j}=U\left(0,1\right)\times \left(UB_{j}-LB_{j}\right)+LB_{j}$$

(5) $$PD_{i,j}=U\left(0,1\right)\times \left(ub_{j}-lb_{j}\right)+lb_{j}$$

In Eqs. (6), (7), $UB_{j}$ and $LB_{j}$ are the upper and lower limitations of the search range. $ub_{j},lb_{j}$, and U(0,1) are uniform distribution random values within [0,1].

(6) $$ub_{j}=\frac{UB_{j}}{m}$$

(7) $$lb_{j}=\frac{LB_{j}}{m}$$

The dog position's fitness function (FF) value is evaluated and stored in the matrix, as shown in Eq. (8). The small value of the FF is pointed and captured as the optimum solution.

(8) $$f\left(PD\right)=\left[\begin{array}{lllll} f_{1}[PD_{1,1} & PD_{1,2} & \cdots & PD_{1,d-1} & \left(PD_{1,d}\;]\right)\\ f_{2}[PD_{2,1} & PD_{2,2} & \cdots & PD_{2,d-l} & \left(PD_{2,d}\;]\right)\\ \vdots & \vdots & PD_{i,j} & \vdots & \vdots \\ f_{n}[PD_{n,1} & PD_{n,2} & \cdots & PD_{n,d-1} & \left(PD_{n,d}\;]\right) \end{array}\right]$$

#### 3.2.2 Exploration phase

The burrow constructing and food search activity is used in this stage. The PDs search for new food sources and create novel burrows around them; they search the group to discover new food sources once the food source is done. The PDs in the colony live in CTs, seek food, and build burrows within the bounds of CT. Here, the maximal iteration counter is split into $\left(iter<\frac{{\mathrm{Max}}_{iter}}{4},\frac{{\;\mathrm{Max}}_{iter}}{4}\leq iter<\frac{{\mathrm{Max}}_{iter}}{2},\frac{{\;\mathrm{Max}}_{iter}}{2}\leq iter\;<3\frac{{\mathrm{Max}}_{iter}}{4},and\;3\frac{{\mathrm{Max}}_{icer}}{4}\leq iter<{\mathrm{Max}}_{iter}\right)$; the first dual arrays are applied for the exploration stage, whereas remaining for the exploitation stage. Eq. (9) demonstrates the updated location for seeking new food sources where the Levy fight (LF) defines the PDs movement. After exploring the food sources, the burrows are constructed near the food sources. The digging strength limits the number of burrows built and Eqs. (9), (10) specify the position update of dogs.

(9) $$PD_{i+1,j+1}=GBest_{i,j}-eCBest_{i,j}\times \rho -CPD_{i,j}\times Levy\left(n\right)\forall iter<\frac{{\mathrm{Max}}_{iter}}{4}$$

(10) $$PD_{i+1,j+1}=GBest_{i,j}\times rPD\times DS\times Levy\left(n\right)F\frac{Max_{iter}}{4}\leq iter<\frac{{\mathrm{Max}}_{iter}}{2}$$

Here, $GBesr_{ij}$ is the global best solution, $eCBesr_{ii}$ is the impact of the current optimum, q is the respective sign for the food source, rPD signifies the position of the random solution, and $CPD_{i,j}$ indicates the random cumulative effect of each member. According to Eqs. (11), (12), (13), the digging power of CT is DS, which is dependent upon the fitness of the food source and is evaluated at random. Levy(n) shows the distribution of Levy for efficient exploration.

(11) $$eCBest_{i,j}=GBest_{i,j}\times \Delta +\frac{PD_{i,j}\times mean\left(PD_{n,m}\right)}{GBest_{i,j}\times \left(UB_{j}-LB_{j}\right)+\Delta }$$

(12) $$CPD_{i,j}=\frac{GBest_{i,j}-rPD_{i,j}}{GBest_{i,j}+\Delta }$$

(13) $$DS=1.5\times r\times {\left(1-\frac{iter}{{\mathrm{Max}}_{iter}}\right)}^{\left(2\frac{iter}{Max_{iter}}\right)}$$

where r refers to arbitrary value in [−1,1]. The factor Δ defines the individual alterations among the members of colonies. Iter and ${\mathrm{Max}}_{iter}$ are the existing and overall iteration counters.

#### 3.2.3 Exploitation phase

The PD produces different scenarios, ranging from the predator menace to the food sources. Communication skills perform a substantial function in fulfilling the need for anti-predation abilities and nutrition. These scenarios adopted in the exploitation stage are shown in Eqs. (14), (15).

$$PD_{i+1,j+1}=GBest_{i,j}-eCBest_{i,j}\times \varepsilon$$

(14) $$-CPD_{i,j}\times rand\forall \frac{{\mathrm{Max}}_{iter}}{2}\leq iter<3\frac{{\mathrm{Max}}_{iter}}{4}$$

(15) $$PD_{i+1,j+1}=GBest_{i,j}\times PE\times rand\forall 3\frac{{\mathrm{Max}}_{iter}}{4}\leq iter<{\mathrm{Max}}_{iter}$$

Now, PE shows the impact of the predator, ε indicates the small number, describing the quality of the food source, and rand is a random integer within [0,1], as illustrated in Eq. (16).

(16) $$PE=1.5\times r\times {\left(1-\frac{iter}{{\mathrm{Max}}_{i;er}}\right)}^{\left(2\frac{iter}{{\mathrm{Max}}_{iter}}\right)}$$

The PDO method improves an FF to attain enhanced classifier performance. It designates an optimistic number to indicate the amended presentation of the candidate solution. In this research, the classifier rate of error minimization has been measured as FF assumed in Eq. (17).

(17) $$fitness\left(x_{i}\right)=ClassifierErrorRate\left(x_{i}\right)=\frac{No.\;of\;misclassified\;instances}{Total\;no.\;of\;instances}\times 100$$

### 3.3 Image classification: CVAE model

Finally, the PDODL-AICA technique uses the CVAE model to detect and classify aerial images. VAE model is a conventional AE network [24]. An encoder and a decoder are combined neural networks of VAE. As an inference model q(z|x), the encoder maps input dataset x to low dimensional hidden variable space z. On the other hand, the decoder obtains the hidden space z variable as the input and output probability of data p(x|z). VAE varies from AE to form a hidden vector; instead of enlarging the bordering log‐likelihood and immediately producing a hidden vector, a vector of the mean (μ) and standard deviation (σ) is generated and combined to create the hidden vector. But, if the network cannot acquire the spreading of those outcomes from the encoder, then the straight integration of this parameter in the hidden vector z represents the constant random variable. The re-parameterization method solves these issues and defines the random variable z as deterministic variables, with z based on the parameter of encoder output (μ;σ) and variable epsilon experimented from the Gaussian distribution shown in Eq. (18):

(18) $$z^{\left(i,l\right)}=\mu^{\left(i\right)}+\sigma^{\left(i\right)}⊙\varepsilon^{\left(l\right)},\varepsilon^{\left(l\right)}N\left(0,I\right)$$

Further, VAE varies from conventional AE in increasing evidence lower bound (ELBO) on minimal log‐likelihood of p(x). The Kullback‐Leibler (KL) difference between the encoder's distribution q(z|x) and the prior distribution p(z) is represented as KL(q(z|x)‖p(z)). Regularization assesses the data lost when p is referred to as distribution q, as depicted in Eq. (19).

(19) $$\min_{p}E_{q\left(z|x\right)}\left[\log\;p\left(x|z\right)\right]-KL\left(q\left(z|x\right)\|p\left(z\right)\right)$$

CVAE is a VAE. The conditional data y is presented as an encoder and decoder model. This creates the sample class to be planned into the encoder's hidden space z, which enhances the ability to discriminate between sample classes and is shown in Eq. (20).

(20) $$\min_{p}\;E_{q\left(z|x,y\right)}\left[\;\log\;p\left(x|z,y\right)\right]-KL\left(q\left(z|x,y\right)\|p\left(z|y\right)\right)$$

**Fig. 2:**
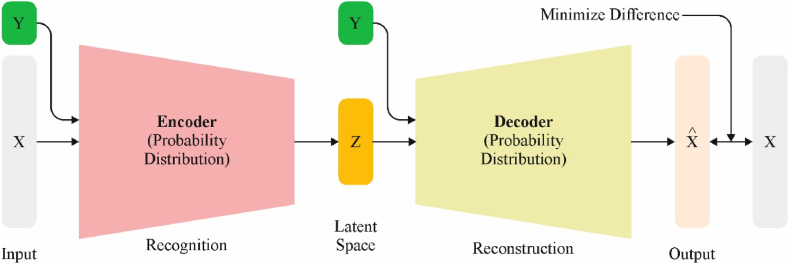
Structure of CVAE.

The data flow of CVAE: the input (x and y) of the encoder, the intermediate hidden space z generated by the network's output, the distribution epsilon, mean (μ), and standard deviation (σ). Fig. 2 demonstrates the architecture of CVAE.

## 4 Experimental validation

**Fig. 3:**
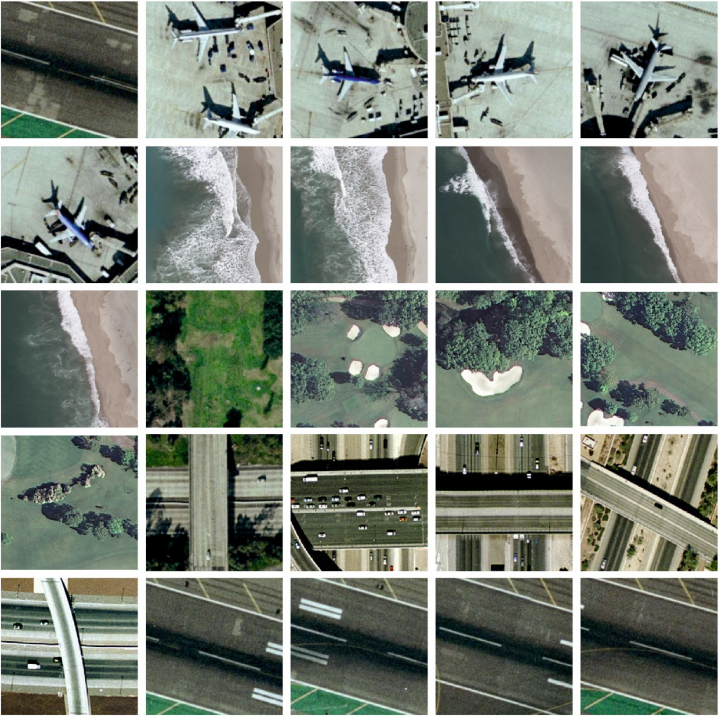
Sample images.

The experimental validation of the PDODL-AICA technique is tested by employing the UCM Merced dataset [25]. The dataset covers an overall of 2100 pictures and 21 classes (mobilehomepark, tenniscourt, agricultural, baseballdiamond, airplane, golfcourse, beach, harbor, sparseresidential, river, intersection, parkinglot, freeway, mediumresidential, buildings, forest, chaparral, storagetanks, overpass, denseresidential and runway) as definite in Table 1. Fig. 3 reveals the sample pictures.

**Table 1:** Details on database.

Classes	Labels	No. of Samples
agricultural	0	100
airplane	1	100
baseballdiamond	2	100
beach	3	100
buildings	4	100
chaparral	5	100
denseresidential	6	100
forest	7	100
freeway	8	100
golfcourse	9	100
harbor	10	100
intersection	11	100
mediumresidential	12	100
mobilehomepark	13	100
overpass	14	100
parkinglot	15	100
river	16	100
runway	17	100
sparseresidential	18	100
storagetanks	19	100
tenniscourt	20	100
**Total No. of Samples**		**2100**

**Fig. 4:**
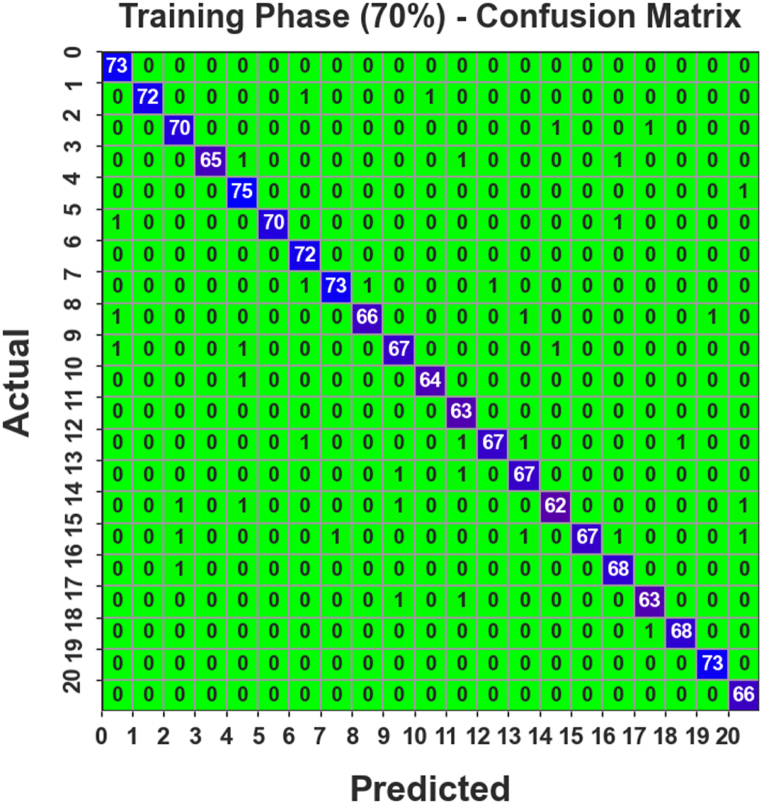
Confusion matrix of PDODL-AICA technique under 70 % of TRAPH.

Fig. 4 establishes the confusion matrix moulded by the AOAFS-HDLCP method below 70 % of TRAPH. The outcomes specified that the PDODL-AICA method correctly categorized 21 classes.

In Table 2, the overall AIC outcomes of the PDODL-AICA technique are reported with 70 % of TRAPH. The results stated that the PDODL-AICA technique correctly classified 21 classes. On class 0, the PDODL-AICA technique offers an $accu_{y}$, $prec_{n}$, $reca_{l}$, $F_{score}$, and $G_{mean}$ of 99.80 %, 96.05 %, 100.00 %, 97.99 %, and 98.01 %, respectively. Additionally, in class 10, the PDODL-AICA methodology provides an $accu_{y}$, $prec_{n}$, $reca_{l}$, $F_{score}$, and $G_{mean}$ of 99.86 %, 98.46 %, 98.46 %, 98.46 %, and 98.46 %, correspondingly. Meanwhile, in class 15, the PDODL-AICA methodology delivers an $accu_{y}$, $prec_{n}$, $reca_{l}$, $F_{score}$, and $G_{mean}$ of 99.66 %, 100.00 %, 93.06 %, 96.40 %, and 96.41 %, respectively. Finally, in class 20, the PDODL-AICA technique offers an $accu_{y}$, $prec_{n}$, $reca_{l}$, $F_{score}$, and $G_{mean}$ of 99.80 %, 95.65 %, 100.00 %, 97.78 %, and 97.80 %, respectively.

**Table 2:** AIC outcome of PDODL-AICA technique under 70 % of TRAPH.

Class Labels	$Accu_{y}$	$Prec_{n}$	$Reca_{l}$	$F_{Score}$	$G_{Mean}$
Training Phase (70 %)					
0	99.80	96.05	100.00	97.99	98.01
1	99.86	100.00	97.30	98.63	98.64
2	99.66	95.89	97.22	96.55	96.55
3	99.80	100.00	95.59	97.74	97.77
4	99.66	94.94	98.68	96.77	96.79
5	99.86	100.00	97.22	98.59	98.60
6	99.80	96.00	100.00	97.96	97.98
7	99.73	98.65	96.05	97.33	97.34
8	99.73	98.51	95.65	97.06	97.07
9	99.59	95.71	95.71	95.71	95.71
10	99.86	98.46	98.46	98.46	98.46
11	99.73	94.03	100.00	96.92	96.97
12	99.66	98.53	94.37	96.40	96.43
13	99.66	95.71	97.10	96.40	96.41
14	99.59	96.88	93.94	95.38	95.40
15	99.66	100.00	93.06	96.40	96.47
16	99.73	95.77	98.55	97.14	97.15
17	99.73	96.92	96.92	96.92	96.92
18	99.86	98.55	98.55	98.55	98.55
19	99.93	98.65	100.00	99.32	99.32
20	99.80	95.65	100.00	97.78	97.80
**Average**	**99.75**	**97.38**	**97.35**	**97.34**	**97.35**

**Fig. 5:**
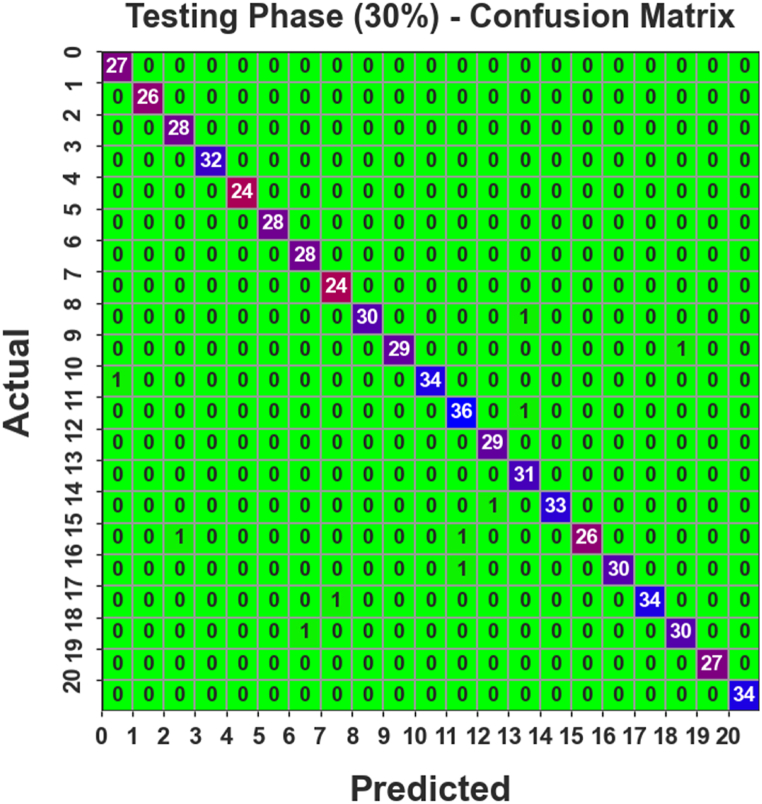
Confusion matrix of PDODL-AICA technique under 30 % of TESPH.

Fig. 5 establishes the confusion matrix formed by the AOAFS-HDLCP method below 30 % of TESPH. The outcomes stated that the PDODL-AICA procedure correctly classified 21 classes.

Table 3 states the complete AIC results of the PDODL-AICA method with 30 % of TESPH. The results specified that the PDODL-AICA method appropriately classified 21 classes. On class 0, the PDODL-AICA approach delivers an $accu_{y}$, $prec_{n}$, $reca_{l}$, $F_{score}$, and $G_{mean}$ of 99.84 %, 96.43 %, 100.00 %, 98.18 %, and 98.20 %, respectively. Moreover, in class 10, the PDODL-AICA approach delivers an $accu_{y}$, $prec_{n}$, $reca_{l}$, $F_{score}$, and $G_{mean}$ of 99.84 %, 100.00 %, 97.14 %, 98.55 %, and 98.56 %, respectively. Meanwhile, in class 15, the PDODL-AICA model provides an $accu_{y}$, $prec_{n}$, $reca_{l}$, $F_{score}$, and $G_{mean}$ of 99.68 %, 100.00 %, 92.86 %, 96.30 %, and 96.36 %, respectively. Lastly, in class 20, the PDODL-AICA model delivers an $accu_{y}$, $prec_{n}$, $reca_{l}$, $F_{score}$, and $G_{mean}$ of 100.00 %, 100.00 %, 100.00 %, 100.00 %, and 100.00 %, respectively.

**Table 3:** AIC outcome of PDODL-AICA technique under 30 % of TESPH.

Class Labels	$Accu_{y}$	$Prec_{n}$	$Reca_{l}$	$F_{Score}$	$G_{Mean}$
Testing Phase (30 %)					
0	99.84	96.43	100.00	98.18	98.20
1	100.00	100.00	100.00	100.00	100.00
2	99.84	96.55	100.00	98.25	98.26
3	100.00	100.00	100.00	100.00	100.00
4	100.00	100.00	100.00	100.00	100.00
5	100.00	100.00	100.00	100.00	100.00
6	99.84	96.55	100.00	98.25	98.26
7	99.84	96.00	100.00	97.96	97.98
8	99.84	100.00	96.77	98.36	98.37
9	99.84	100.00	96.67	98.31	98.32
10	99.84	100.00	97.14	98.55	98.56
11	99.52	94.74	97.30	96.00	96.01
12	99.84	96.67	100.00	98.31	98.32
13	99.68	93.94	100.00	96.88	96.92
14	99.84	100.00	97.06	98.51	98.52
15	99.68	100.00	92.86	96.30	96.36
16	99.84	100.00	96.77	98.36	98.37
17	99.84	100.00	97.14	98.55	98.56
18	99.68	96.77	96.77	96.77	96.77
19	100.00	100.00	100.00	100.00	100.00
20	100.00	100.00	100.00	100.00	100.00
**Average**	**99.85**	**98.46**	**98.50**	**98.45**	**98.47**

**Fig. 6:**
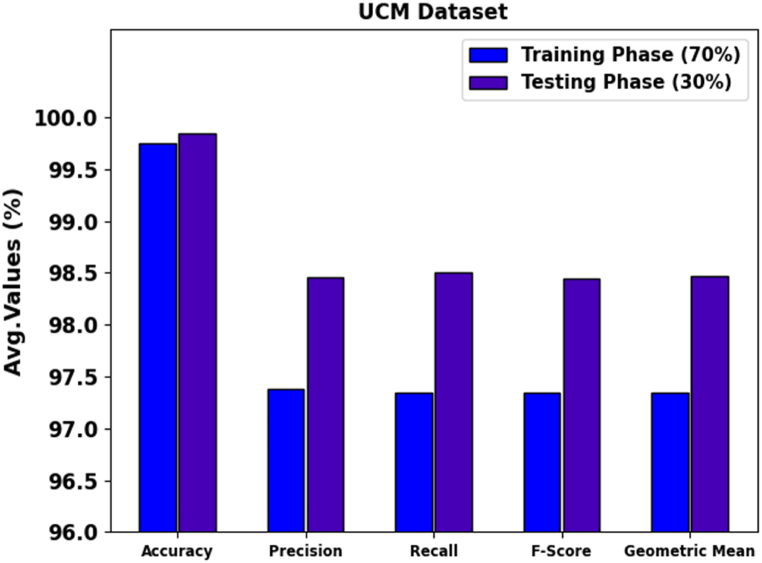
Average of PDODL-AICA technique under 70:30 of TRAPH/TESPH.

Fig. 6 shows the average AIC results of the PDODL-AICA technique. The results highlighted that the PDODL-AICA technique correctly identified 21 classes. With 70 % of TRAPH, the PDODL-AICA technique provides an average $accu_{y}$, $prec_{n}$, $reca_{l}$, $F_{score}$, and $G_{mean}$ of 99.75 %, 97.38 %, 97.35 %, 97.34 %, and 97.35 %, respectively. In addition, with 30 % of TESPH, the PDODL-AICA method delivers an average $accu_{y}$, $prec_{n}$, $reca_{l}$, $F_{score}$, and $G_{mean}$ of 99.85 %, 98.46 %, 98.50 %, 98.45 %, and 98.47 %, correspondingly.

**Fig. 7:**
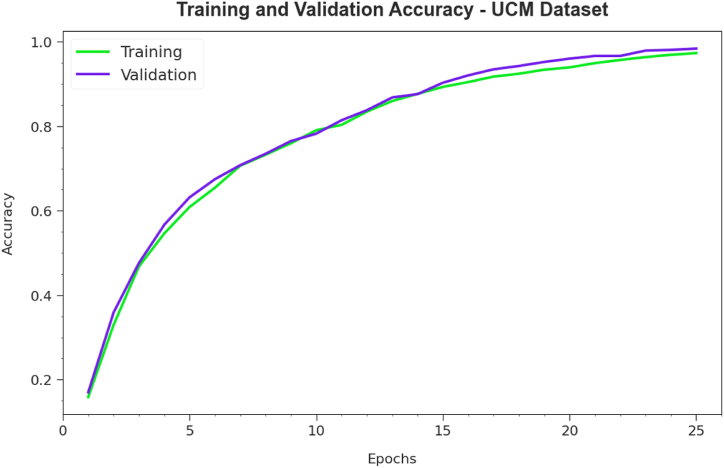
$Accu_{y}$ the curve of the PDODL-AICA technique under the UCM dataset.

The $accu_{y}$ curves for training (TRA) and validation (VL) exposed in Fig. 7 for the PDODL-AICA technique under the UCM dataset deliver valued insights into its performance under numerous epochs. Mainly, there is a consistent development in both TRA and TES $accu_{y}$ to rising epochs, demonstrating the model's capability to acquire and recognize designs from both TRA and TES data. The growing trend in TES $accu_{y}$ underlines the model's flexibility in relation to the TRA dataset and its ability to generate precise predictions on hidden data, underlining strong generalization skills.

**Fig. 8:**
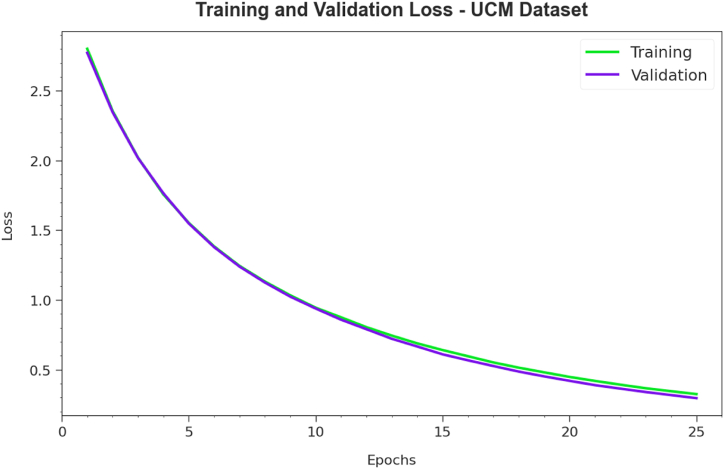
Loss curve of PDODL-AICA technique under UCM dataset.

Fig. 8 summarises the TRA and TES loss values for the PDODL-AICA technique below the UCM dataset across several epochs. The TRA loss reliably decreases as the model improves its weights to minimize classification error on both datasets. The loss curves demonstrate the model's arrangement with the TRA data, underlining its capability to take designs well in both datasets. Notable is the nonstop alteration of parameters in the PDODL-AICA technique, which is planned to minimize differences between forecasts and real TRA labels.

**Fig. 9:**
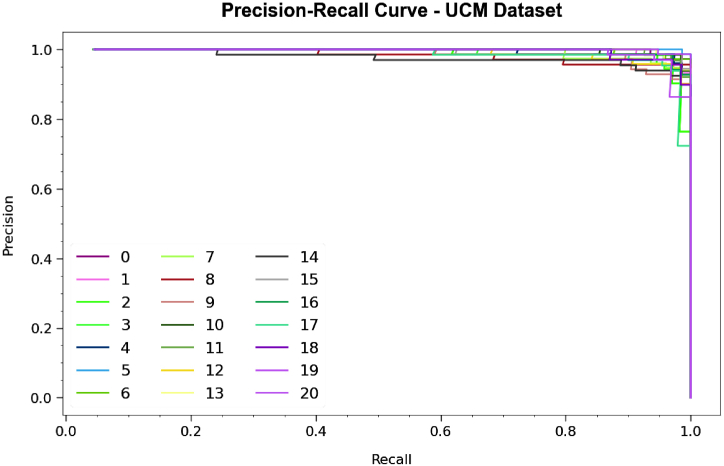
PR curve of PDODL-AICA technique under UCM dataset.

Regarding the precision-recall (PR) curve in Fig. 9, the results confirm that the PDODL-AICA method below the UCM dataset reliably attains enhanced PR values across every class. These outcomes underline the model's real capacity for discriminating amongst diverse classes, underlining its value in accurately in class labels.

**Fig. 10:**
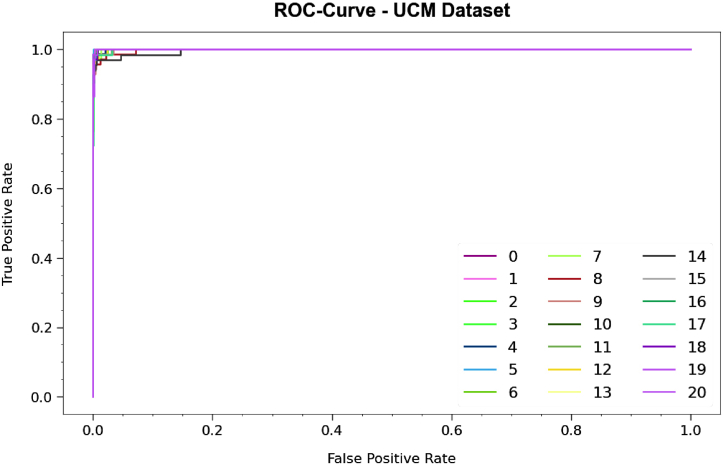
ROC curve of PDODL-AICA technique under UCM dataset.

Besides, in Fig. 10, ROC curves produced by the PDODL-AICA method are presented below the UCM dataset, representing its ability to differentiate among classes. These curves deliver valuable insights into how TPR and FPR tradeoffs differ across dissimilar classification epochs and thresholds. The results underline the model's exact classification performance under several class labels, underlining its efficiency in addressing various classification tasks.

**Fig. 11:**
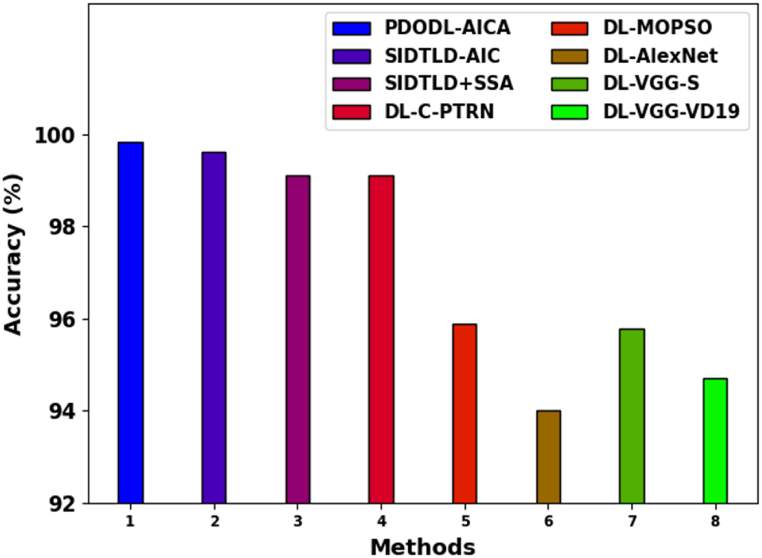
$Accu_{y}$ analysis of the PDODL-AICA technique with other approaches.

Table 4 reports a detailed comparison study of the PDODL-AICA model under distinct measures [27]. In Fig. 11, a comparative $accu_{y}$ analysis of the PDODL-AICA technique is given. The results indicate that the DL-MOPSO, DL-AlexNet, DL-VGG-S, and DL-VGG-VD19 techniques have performed worse than other approaches. The SIDTLD-AIC, SIDTLD + SSA, and DL-C-PTRN techniques also accomplish closer classification performance. However, the PDODL-AICA technique exhibits superior performance with a maximum $accu_{y}$ of 99.85 %.

**Table 4:** Comparative analysis of PDODL-AICA technique with other approaches.

Methods	$Accu_{y}$	$Prec_{n}$	$Reca_{l}$	$F_{Score}$	$G_{Mean}$
PDODL-AICA	99.85	98.46	98.5	98.45	98.47
SIDTLD-AIC	99.62	95.90	95.87	95.87	97.81
SIDTLD + SSA	99.12	93.97	94.31	94.48	93.15
DL-C-PTRN	99.11	93.25	92.92	93.47	93.58
DL-MOPSO	95.90	94.27	95.10	93.62	93.90
DL-AlexNet	94.02	94.02	93.49	94.63	93.16
DL-VGG-S	95.78	93.16	94.49	93.03	93.07
DL-VGG-VD19	94.71	94.11	94.42	94.91	94.96

**Fig. 12:**
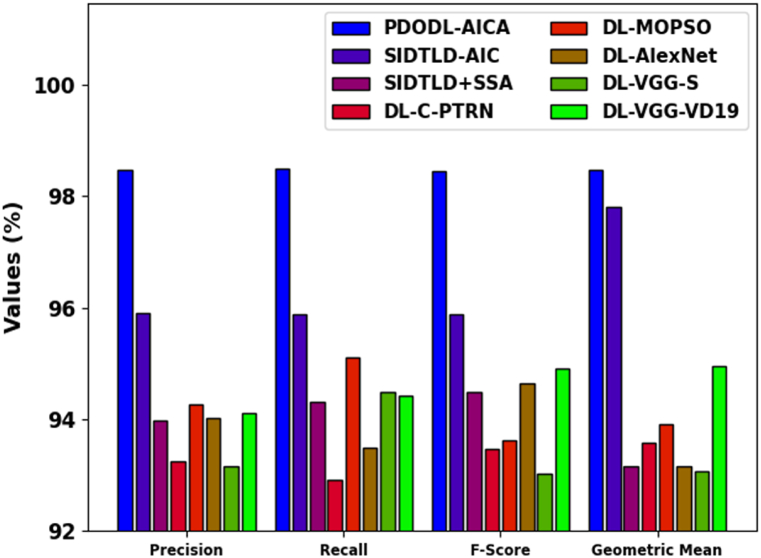
Comparative analysis of PDODL-AICA technique with other approaches.

In Fig. 12, a comparative $prec_{n}$, $reca_{l}$, $F_{score}$, and $G_{mean}$ study of the PDODL-AICA model is given. The results specify that the DL-MOPSO, DL-AlexNet, DL-VGG-S, and DL-VGG-VD19 approaches have exposed worse performance over other techniques. Besides that, the SIDTLD-AIC, SIDTLD + SSA, and DL-C-PTRN methods achieve nearer classification performance. But, the PDODL-AICA model shows higher performance with the greatest $prec_{n}$ of 98.46 %, $reca_{l}$ of 98.5 %, $F_{score}$ of 98.45 %, $\text{and}\;G_{mean}$ of 98.47 %.

**Fig. 13:**
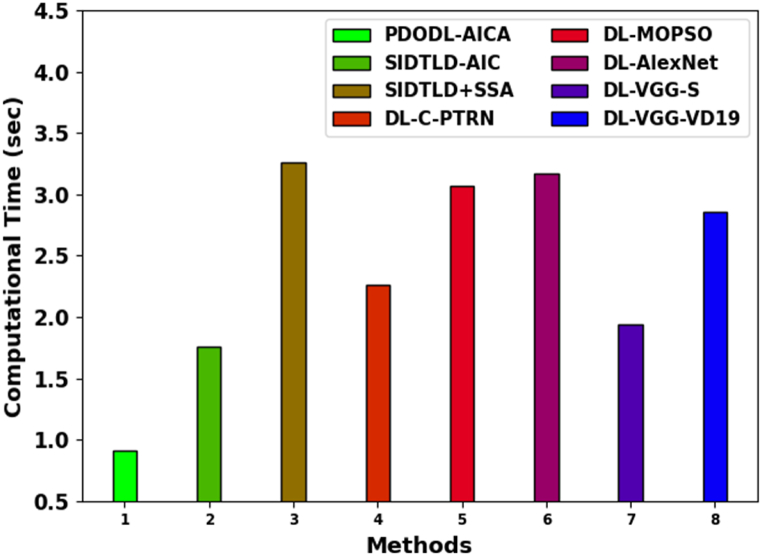
CT analysis of PDODL-AICA technique with other approaches.

Table 5 and Fig. 13 comprehensively compare the computational time (CT) study of the PDODL-AICA method.

**Table 5:** CT analysis of PDODL-AICA technique with other approaches.

Methods	Computational Time (sec)
PDODL-AICA	0.91
SIDTLD-AIC	1.76
SIDTLD + SSA	3.26
DL-C-PTRN	2.26
DL-MOPSO	3.07
DL-AlexNet	3.17
DL-VGG-S	1.94
DL-VGG-VD19	2.86

The outcomes specify that the DL-MOPSO, DL-AlexNet, DL-VGG-S, and DL-VGG-VD19 techniques have revealed worse performance than other techniques. Besides that, the SIDTLD-AIC, SIDTLD + SSA, and DL-C-PTRN approaches achieve closer classification performance. However, the PDODL-AICA model performs better with a smaller CT of 0.91s. These superior results stated the enhanced performance of the PDODL-AICA technique over other recent methods in terms of distinct measures.

## 5 Conclusion

In this study, a novel PDODL-AICA technique on UAV images is presented. The PDODL-AICA technique exploits the optimal DL model for classifying aerial images into manifold classes. The PDODL-AICA technique contains an EfficientNetB7-based feature extraction, PDO-based parameter tuning, and CVAE-based classification processes. At an early phase, the presented PDODL-AICA model is achieved using the EfficientNetB7 technique for the feature extractor process. Besides, the hyperparameter tuning of the EfficientNetB7 technique uses the PDO model. The PDODL-AICA technique uses the CVAE model to detect and classify aerial images. The performance study of the PDODL-AICA model is implemented on a benchmark UAV image dataset. The experimental values inferred the authority of the PDODL-AICA model over recent approaches in terms of dissimilar methods. The limitations of the PDODL-AICA technique comprise computational complexity due to the deep architecture and the requirement for crucial computational resources. Future studies should focus on optimizing the effectualness of the method for real-time applications and addressing scalability problems. Furthermore, exploring models to improve the interpretability of CVAE-generated features and enhancing robustness against various convolutional real-world scenarios would be significant for advancing the technique's applicability in practical scenarios.

## Data availability statement

The data supporting this study's findings are available in the UCM Merced dataset, reference number [25].

## CRediT authorship contribution statement

**Amal K. Alkhalifa:** Writing – original draft, Methodology, Investigation, Conceptualization. **Muhammad Kashif Saeed:** Formal analysis, Data curation. **Kamal M. Othman:** Resources, Project administration, Methodology. **Shouki A. Ebad:** Writing – review & editing, Resources, Project administration, Funding acquisition. **Mohammed Alonazi:** Supervision, Software, Resources. **Abdullah Mohamed:** Visualization, Validation, Supervision.

## Declaration of competing interest

The authors declare that they have no known competing financial interests or personal relationships that could have appeared to influence the work reported in this paper.

The authors declare the following financial interests/personal relationships which may be considered as potential competing interests:

All authors have participated in (a) conception and design, or analysis and interpretation of the data; (b) drafting the article or revising it critically for important intellectual content; and (c) approval of the final version.

This manuscript has not been submitted to, nor is under review at, another journal or other publishing venue.

## References

[1] Abunadi I., Althobaiti M.M., Al-Wesabi F.N., Hilal A.M., Medani M., Hamza M.A., Rizwanullah M., Zamani A.S. (2022). Ederated learning with blockchain assisted image classification for clustered UAV networks. Comput. Mater. Continua (CMC).

[2] Li J., Yan D., Luan K., Li Z., Liang H. (2020). Deep learning-based bird's nest detection on transmission lines using UAV imagery. Appl. Sci..

[3] Youme O., Bayet T., Dembele J.M., Cambier C. (2021). Deep learning and remote sensing: detection of dumping waste using UAV. Procedia Comput. Sci..

[4] Mittal P., Singh R., Sharma A. (2020). Deep learning-based object detection in low-altitude UAV datasets: a survey. Image Vis Comput..

[5] Bashmal L., Bazi Y., Al Rahhal M.M., Alhichri H., Al Ajlan N. (2021). UAV image multi-labeling with data-efficient transformers. Appl. Sci..

[6] Anwer M.H., Hadeel A., Fahd N.A.-W., Mohamed K.N., Abdelwahed M., Anil K., Ishfaq Y., Abu Sarwar Z. (2022). Fuzzy cognitive maps with bird swarm intelligence optimization-based remote sensing image classification. Comput. Intell. Neurosci..

[7] Tetila E.C., Machado B.B., Astolfi G., de Souza Belete N.A., Amorim W.P., Roel A.R., Pistori H. (2020). Detection and classification of soybean pests using deep learning with UAV images. Comput. Electron. Agric..

[8] Öztürk A.E., Erçelebi E. (2021). Real UAV-bird image classification using CNN with a synthetic dataset. Appl. Sci..

[9] Ammour N., Alhichri H., Bazi Y., Benjdira B., Alajlan N., Zuair M. (2017). Deep learning approach for car detection in UAV imagery. Rem. Sens..

[10] Behera T.K., Bakshi S., Nappi M., Sa P.K. (2023). Superpixel-based multiscale CNN approach toward multiclass object segmentation from UAV-captured aerial images. IEEE J. Sel. Top. Appl. Earth Obs. Rem. Sens..

[11] Rahman A.K.Z.R., Sakif S., Sikder N., Masud M., Aljuaid H., Bairagi A.K. (2023). Unmanned aerial vehicle assisted forest fire detection using deep convolutional neural network. Intell. Autom. Soft Comput.

[12] Dewangan O., Vij P. (2024).

[13] Behera T.K., Bakshi S., Sa P.K. (2022). Vegetation extraction from UAV-based aerial images through deep learning. Comput. Electron. Agric..

[14] Pandey A., Jain K. (2022). An intelligent system for crop identification and classification from UAV images using conjugated dense convolutional neural network. Comput. Electron. Agric..

[15] Jiskani S.M., Hussain T., Sahito A.A., Shaikh F., Shah A.A. (2024). Aerial identification of flashed over faulty insulator using binary image classification. Mehran Univ. Res. J. Eng. Technol..

[16] Minu M.S., Canessane R.A. (2022). Deep learning-based aerial image classification model using inception with residual network and multilayer perceptron. Microprocess. Microsyst..

[17] Samadzadegan F., Dadrass Javan F., Ashtari Mahini F., Gholamshahi M. (2022). Detection and recognition of drones based on a deep convolutional neural network using visible imagery. Aerospace.

[18] Cheng G., Xie X., Han J., Guo L., Xia G.S. (2020). Remote sensing image scene classification meets deep learning: challenges, methods, benchmarks, and opportunities. IEEE J. Sel. Top. Appl. Earth Obs. Rem. Sens..

[19] Geetha N., Sunitha G. (2024). Pelican optimization algorithm with convolutional-recurrent hop field neural network for unmanned aerial image classification model. Multimed. Tool. Appl..

[20] Mogaka O.M., Zewail R., Inoue K., Sayed M.S. (2024). TinyEmergencyNet: a hardware-friendly ultra-lightweight deep learning model for aerial scene image classification. Journal of Real-Time Image Processing.

[21] Raza R., Zulfiqar F., Khan M.O., Arif M., Alvi A., Iftikhar M.A., Alam T. (2023). Lung-EffNet: lung cancer classification using EfficientNet from CT-scan images. Eng. Appl. Artif. Intell..

[22] Sultan H.M., Menesy A.S., Alqahtani M., Khalid M., Diab A.A.Z. (2023). Accurate parameter identification of proton exchange membrane fuel cell models using different metaheuristic optimization algorithms. Energy Rep..

[23] Abdellatif A., Mubarak H., Abdellatef H., Kanesan J., Abdelltif Y., Chow C.O., Chuah J.H., Gheni H.M., Kendall G. (2024). Computational detection and interpretation of heart disease based on conditional variational auto-encoder and stacked ensemble-learning framework. Biomed. Signal Process Control.

[24] http://weegee.vision.ucmerced.edu/datasets/landuse.html.

[25] S. Alotaibi S., Abdullah Mengash H., Negm N., Marzouk R., Hilal A.M., Shamseldin M.A., Motwakel A., Yaseen I., Rizwanullah M., Zamani A.S. (2022). Swarm intelligence with deep transfer learning driven aerial image classification model on UAV networks. Appl. Sci..

